# Evidence for spatial clines and mixed geographic modes of speciation for North American cherry‐infesting *Rhagoletis* (Diptera: Tephritidae) flies

**DOI:** 10.1002/ece3.6667

**Published:** 2020-10-28

**Authors:** Meredith M. Doellman, Gilbert Saint Jean, Scott P. Egan, Thomas H. Q. Powell, Glen R. Hood, Hannes Schuler, Daniel J. Bruzzese, Mary M. Glover, James J. Smith, Wee L. Yee, Robert Goughnour, Juan Rull, Martin Aluja, Jeffrey L. Feder

**Affiliations:** ^1^ Department of Biological Sciences University of Notre Dame Notre Dame Indiana USA; ^2^ Advanced Diagnostics & Therapeutics University of Notre Dame Notre Dame Indiana USA; ^3^ Department of BioSciences Rice University Houston Texas USA; ^4^ Department of Biological Sciences Binghamton University Binghamton New York USA; ^5^ Department of Biological Sciences Wayne State University Detroit Michigan USA; ^6^ Faculty of Science and Technology Free University of Bozen‐Bolzano Bozen Italy; ^7^ Department of Entomology Lyman Briggs College Michigan State University East Lansing Michigan USA; ^8^ Temperate Tree Fruit & Vegetable Research Unit United States Department of Agriculture, Agricultural Research Service Wapato Washington USA; ^9^ Washington State University Extension Vancouver Washington USA; ^10^ Instituto de Ecología, A.C. Xalapa México; ^11^ LIEMEN‐División Control Biológico de Plagas PROIMI Biotecnología‐CONICET Tucumán Argentina; ^12^ Environmental Change Initiative University of Notre Dame Notre Dame Indiana USA; ^13^ Present address: Department of Ecology and Evolution University of Chicago Chicago Illinois USA

**Keywords:** allopatry, climate change, isolation by distance, microsatellites, mtDNA, range fragmentation, *Rhagoletis cingulata*, *Rhagoletis indifferens*, wing spot

## Abstract

An important criterion for understanding speciation is the geographic context of population divergence. Three major modes of allopatric, parapatric, and sympatric speciation define the extent of spatial overlap and gene flow between diverging populations. However, mixed modes of speciation are also possible, whereby populations experience periods of allopatry, parapatry, and/or sympatry at different times as they diverge. Here, we report clinal patterns of variation for 21 nuclear‐encoded microsatellites and a wing spot phenotype for cherry‐infesting *Rhagoletis* (Diptera: Tephritidae) across North America consistent with these flies having initially diverged in parapatry followed by a period of allopatric differentiation in the early Holocene. However, mitochondrial DNA (mtDNA) displays a different pattern; cherry flies at the ends of the clines in the eastern USA and Pacific Northwest share identical haplotypes, while centrally located populations in the southwestern USA and Mexico possess a different haplotype. We hypothesize that the mitochondrial difference could be due to lineage sorting but more likely reflects a selective sweep of a favorable mtDNA variant or the spread of an endosymbiont. The estimated divergence time for mtDNA suggests possible past allopatry, secondary contact, and subsequent isolation between USA and Mexican fly populations initiated before the Wisconsin glaciation. Thus, the current genetics of cherry flies may involve different mixed modes of divergence occurring in different portions of the fly's range. We discuss the need for additional DNA sequencing and quantification of prezygotic and postzygotic reproductive isolation to verify the multiple mixed‐mode hypothesis for cherry flies and draw parallels from other systems to assess the generality that speciation may commonly involve complex biogeographies of varying combinations of allopatric, parapatric, and sympatric divergence.

## Graphical abstract

Allopatry is often considered the null model for speciation, but there is often more than meets the eye for many seemingly straight‐forward stories of allopatric divergence. Here, we use patterns of genetic and morphological variation to demonstrate that two currently allopatric sibling species of cherry‐infesting flies likely diverged via a more complex mixed mode of speciation. Discordant nuclear and mitochondrial data suggest important roles for both biogeographic processes and endosymbionts in divergence. These complexities may have ramifications for understanding the evolution of reproductive isolation between populations leading to the formation of new species.

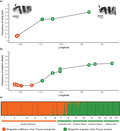

## 1 INTRODUCTION

Biogeography is a key consideration for understanding speciation, as it affects the relationship between geographic isolation and gene flow under which reproductive isolation (RI) evolves and new biodiversity forms (Coyne & Orr, 2004). The geographic context of speciation has traditionally been divided into allopatric, parapatric, and sympatric modes, depending upon the spatial distribution of diverging populations (Bush, 1975; Coyne & Orr, 2004). Under classic allopatric or vicariant speciation (Figure S1a), populations are physically separated (become bifurcated) into two large subpopulations by an uninhabitable area (Mayr, 1963). These isolates subsequently diverge in allopatry to form new species. Parapatric or clinal speciation (Figure S1b) occurs when an ancestral population diverges across a spatial or ecological gradient (Endler, 1977). In this case, populations partly overlap with one another or can be arrayed as a series of stepping‐stones partially connected by gene flow, as they evolve into different species. Finally, sympatric speciation (Figure S1c) occurs when diverging populations completely overlap, such that individuals of the evolving taxa remain in the cruising range of one another (Bush, 1975; Mayr, 1963).

The different modes of speciation are associated with different levels of gene flow among populations during the divergence process. Under allopatric speciation (Figure S1a), gene flow between populations ceases due to a physical barrier of inhospitable habitat preventing migration (Mayr, 1963). The isolated populations are consequently free to independently accumulate genetic differences by natural selection and/or genetic drift to evolve RI, eventually leading to speciation. In parapatric speciation (Figure S1b), the partial overlap of populations results in a reduction but not elimination of migration between diverging demes. In this case, primary clines of changing gene and phenotype frequencies can evolve across the landscape generating a pattern of isolation by distance (IBD), which can potentially result in populations on different sides of the clines evolving into reproductively isolated species (Endler, 1977). Sympatric speciation (Figure S1c) assumes that gene flow is possible throughout the divergence process. In this case, ecologically based divergent selection associated with biotic or abiotic environmental conditions or sexual selection is greater (stronger) than the level of gene flow, permitting populations to differentiate in the absence of geographic isolation (Bush, 1975; Mayr, 1963; Nosil, 2012). Distinguishing among these biogeographic possibilities is important because it can shed light on the factors and chronology whereby different types of RI evolve during speciation.

The classification of speciation into distinct allopatric, parapatric, and sympatric modes is to some degree one of convenience, distinguishing different general relationships between geographic isolation and gene flow during speciation. While it is possible for speciation to occur from start to finish solely in allopatry, parapatry, or sympatry, in reality, this may not always be the case. In other words, mixed or “hybrid” modes of speciation may be common in which the geographic context and levels of gene flow temporally vary during the divergence process (Coyne & Orr, 2004; Xie et al., 2007).

One example of a mixed mode of speciation involves secondary contact (Figure S2a). In this case, a population initially becomes subdivided into isolates that diverge in allopatry. Through time, the physical barrier separating taxa dissipates and populations reestablish contact. If diverged populations have not evolved complete RI, then secondary genetic clines (a hybrid zone) can form, reflecting rates of migration, interbreeding, and selection against hybrids (Barton & Hewitt, 1981, 1985, 1989). Moreover, when a degree of postzygotic isolation has evolved in allopatry, a process of “reinforcement” can occur following secondary contact in which increased prezygotic RI is selected for to reduce the production of less fit hybrids (Servedio & Noor, 2003). Thus, reinforcement following secondary contact represents a mixed mode of speciation in that RI does not evolve solely in allopatry in the absence of gene flow. The same applies to other mechanisms in addition to reinforcement that can evolve following secondary contact to increase RI between populations in the face of gene flow (e.g., divergent ecological adaptation to different habitats). Such a mixed mode has been termed “allo‐parapatric” or “allo‐sympatric” by Coyne and Orr (2004). Populations in secondary contact may reflect a single cycle of allopatry, divergence, and subsequent overlap (Figure S2a). However, populations can potentially undergo repeated cycles of allopatry and secondary contact during their history, with divergence accruing at a variety of different times when populations were isolated and overlapped (Hewitt, 2000). Thus, there can also be various types of multiple mixed modes of speciation, such as “allo‐para‐allopatric,” as the geographic distributions of diverging taxa change repeatedly over time (Figure S2b).

Although there are many examples of secondary contact and hybrid zones in nature (see Barton & Hewitt, 1985; Coyne & Orr, 2004 for synopsis), some of which may involve the evolution of reinforcement (Servedio & Noor, 2003), there are other possible chronologies in which a mixed mode of speciation may unfold. One alternative mixed mode involves divergence beginning in parapatry, with populations initially forming a “primary” cline (Barton & Hewitt, 1981, 1989; Figure S2c). Over time, the populations on the ends of the clines may become geographically isolated, with intermediate populations potentially going extinct. In allopatry, the two populations can subsequently continue diverging and evolve additional RI. The above scenario is mixed because divergence did not accumulate only after the complete geographic isolation of populations but accrued to some degree in the face of gene flow in parapatry (or even sympatry) prior to allopatry. Such a mixed mode has been termed “para‐allopatric” by Coyne and Orr (2004).

Envisioning different types of mixed or multiple mixed modes of speciation are not difficult and population divergence may often occur by such means in nature (Coyne & Orr, 2004). The problem is demonstrating their occurrence, as finding evidence supporting a mixed mode can be challenging, even for the seemingly straight‐forward case of reinforcement following secondary contact (Barton & Hewitt, 1985; Butlin, 1989). In this regard, testing requires a study system having a known biogeography with features allowing inferences to be made that populations diverged under differing spatial contexts (e.g., parapatric and allopatric), experiencing varying levels of gene flow through time in the process. The problem is twofold. First, one must characterize the biogeography of a pair of taxa to demonstrate that it has changed through time. Second, one must show that during times when taxa were both in contact and experiencing gene flow and geographically separated and isolated, they evolved RI.

**FIGURE 1:**
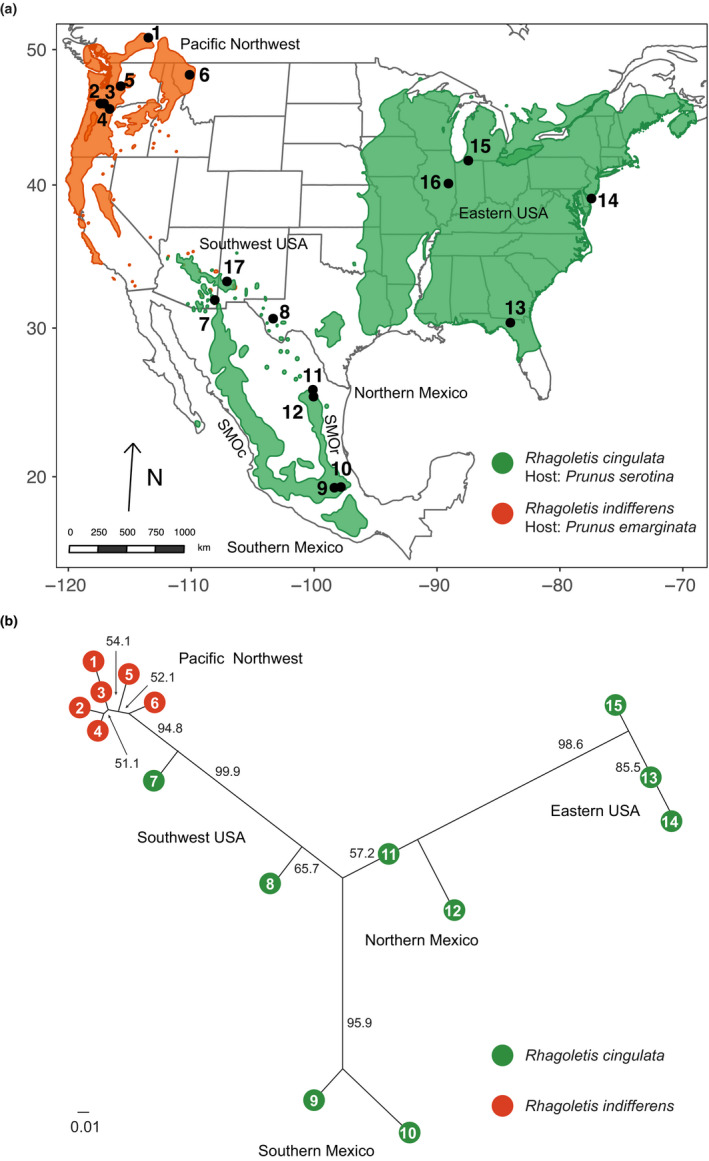
(a) Map of collecting sites (Mollweide projection). Shown are the numbered locations of collecting sites for cherry‐infesting *Rhagoletis* from across North America, as designated in Table S1. The green area indicates the geographic range of *Prunus serotina*, black cherry, and orange area the range of *P. emarginata*, bitter cherry. Note that the two cherry tree species do cooccur in areas of the states of Arizona and New Mexico, where bitter cherry is rare. SMOr = Sierra Madre Oriental; SMOc = Sierra Madre Occidental Mountains in Mexico; (b) Neighbor‐joining genetic distance network for cherry‐infesting *Rhagoletis* across North America based on 21 microsatellite loci. Orange circles represent *R. indifferens* infesting bitter cherry from the Pacific Northwest (PNW), green circles represent *R. cingulata* infesting black cherry from the southwest USA (SW), southern (SoM) and northern Mexico (NoM), and the eastern USA (ENA). Sites 16 and 17 were sequenced for mtDNA only and, thus, do not appear on the network. Also shown are bootstrap support levels > 50%. A Nei's genetic distance of 0.01 is indicated by the scale bar

Here, we focus on the first question of changing biogeography by examining two sister taxa of cherry‐infesting fruit flies in the genus *Rhagoletis* (Diptera: Tephritidae), *Rhagoletis cingulata,* and *Rhagoletis indifferens* that possess attributes conducive for undergoing and testing for a mixed mode of divergence (Bush, 1966; see Supplemental Background for additional details). The current ranges of *R. cingulata* and *R. indifferens* on face value appear to conform to a classic vicariant model of allopatric speciation. Specifically, *R. cingulata* and *R. indifferens* presently have a disjunct distribution, being found in two large and contiguous populations in eastern North America (ENA) and the Pacific Northwest (PNW), respectively, separated by ~1,900 km across the central and northern plain states of the USA and provinces of southcentral Canada (Figure 1a; Bush, 1966). Across the plains, neither the primary host of *R. cingulata* (black cherry, *Prunus serotina* Ehrh) nor of *R. indifferens* (bitter cherry, *P. emarginata* [Dougl. Ex Hook.] Eaton) occurs. In addition, a third geographically isolated population of *R. cingulata* exists on *P. serotina* in southern Mexico (SoM; Figure 1a). Thus, if cherry flies have or are speciating via a single mode of divergence, the appropriate null hypothesis would be an allopatric model reflecting their current geographic isolation and not parapatric or sympatric alternatives.

However, the geographic distribution of cherry flies is more complicated than a simple PNW versus ENA bifurcation or PNW, ENA, and SoM trifurcation. Flies described as *R. cingulata* and their black cherry host plants also exist in a semicircular arc of isolated populations found at higher elevations through the southwestern USA (SW) states of Texas, New Mexico, and Arizona that could have previously connected or been the source of the populations in the PNW and ENA (Figure 1a; Bush, 1966). In these isolated “sky islands” of the SW (Dodge, 1943; Heald, 1967) and northern Mexico (NoM), flies and their black cherry hosts persist in restricted pockets in certain mountain canyons experiencing higher rainfall and lower temperatures than surrounding low‐lying deserts (Bush, 1966). Black cherries and *R. cingulata* also extend southerly from the SW through isolated mountains in the Chihuahuan Desert in NoM down into the Sierra Madre Oriental Mountains (SMOr) and SoM (Figure 1a; Bush, 1966; Rull, Aluja, & Feder, 2011).

**FIGURE 2:**
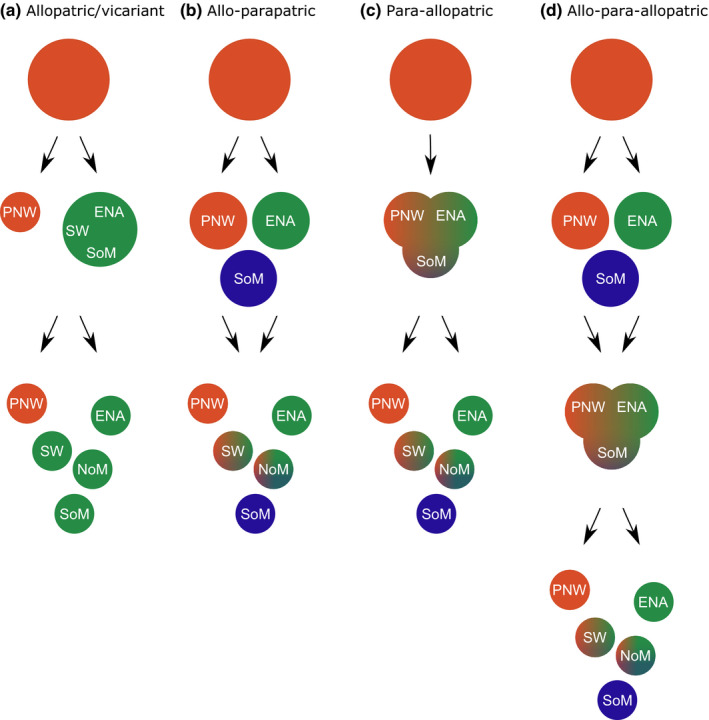
Alternative geographic divergence hypotheses for cherry‐infesting *Rhagoletis* in North America. (a) Vicariance model; a large, ancestral cherry fly population harboring little to no spatial variation bifurcated into *R. cingulata* and *R. indifferens* along a spatial divide between the southwestern USA (SW) and Pacific Northwest (PNW). Subsequently, *R. cingulata* became recently subdivided into Southwestern, northern Mexican (NoM), southern Mexican (SoM), and eastern USA (ENA) subpopulations. Note that the classic allopatric/ vicariant model predicts sharp genetic and phenotypic breaks should currently be seen between cherry flies in the PNW and elsewhere, as indicated by the orange compared to green color, respectively, of populations in these regions; (b) Allo‐parapatric model; a large, ancestral population harboring little to no spatial variation separated into ENA, PNW, and SoM isolates. These isolates subsequently diverge in allopatry. Most recently in the Holocene, flies were reintroduced into the SW and NoM, forming secondary clines; (c) Para‐allopatric model; a primary cline of isolation by distance forms through the ancestral cherry fly population across the SW and NoM sometime during the Wisconsin glaciation. As ice sheets retreated following the last glacial maximum, cherry flies migrated into ENA and the PNW. Subsequent warming and drying in the early Holocene resulted in these populations and those in the SW and NoM becoming geographically isolated and differentiating; (d) Allo‐para‐allopatric model; an ancestral cherry fly population becomes geographically isolated and diverges into ENA, PNW, and SoM subpopulations during the interglacial period preceding the Wisconsin glaciation. Populations then reestablish contact during the Wisconsin glaciation, creating secondary clines through the SW and NoM. In addition, introgression from SoM introduced the diverged mtDNA haplotype II into NoM and the SW. Subsequent warming and drying in the early Holocene resulted in the cherry fly populations becoming geographically separated into PNW, SW, NoM, SoM, and ENA and differentiating, with the mtDNA haplotype II not reaching the PNW and ENA prior to regional subdivision. A multiple mixed model involves a combination of hypotheses c and d, with primary clines forming across the SW and NoM being augmented by allopatry, secondary contact, and gene flow from SoM, especially for mtDNA haplotype II

**FIGURE 3:**
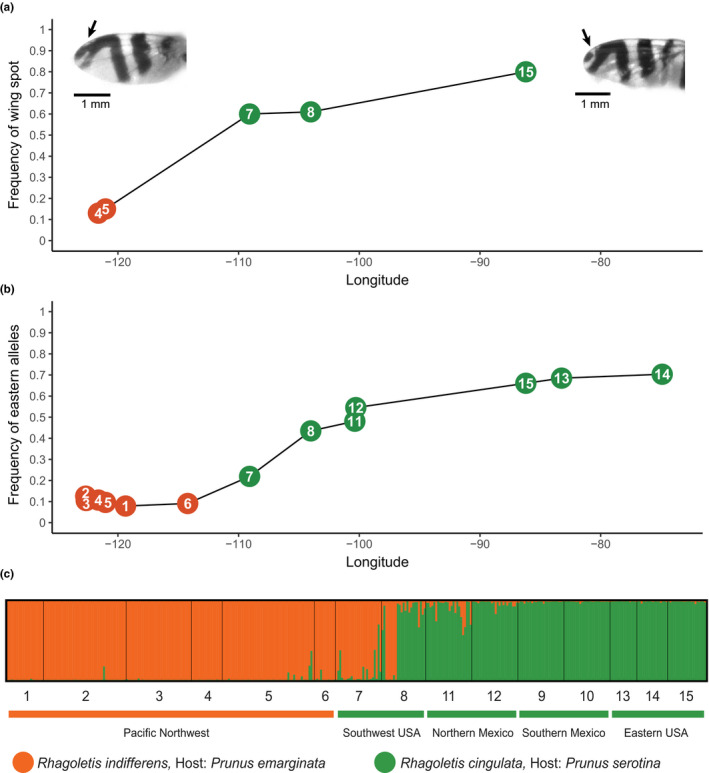
(a) Frequency of the apical wing spot for two cherry‐infesting *Rhagoletis* populations in the Pacific Northwest (PNW sites 4 and 5), two populations in the southwest USA (SW sites 7 and 8), and one population in the eastern USA (ENA site 15) plotted against each population's longitude. Insets display the wing pattern for a *R. cingulata* at site 15 possessing the apical wing spot (right picture designated by arrow) and a *R. indifferens* at site 4 having a solid apical wing band (left picture designated by arrow). A distance of 1 mm is indicated by the scale bar in each inset. (b) Plot of mean microsatellite allele frequency versus longitude for populations across the PNW, SW, northern Mexico (NoM), and ENA for alleles more common in ENA than the PNW for the 21 loci. (c) structure plot for *K* = 2 subpopulations determined to have the highest likelihood by the method of Evanno et al. (2005). See Table S1 for full list of designations and descriptions of study sites and Figure 1a for a map of collecting sites. Sites 1–6 are *R. indifferens* populations in the PNW, sites 7–8 are *R. cingulata* in the SW, sites 9–10 are *R. cingulata* in southern Mexico (SoM), sites 11–12 are *R. cingulata* in NoM, and sites 13–15 *R. cingulata* in ENA

The current distribution of *R. cingulata* and *R. indifferens* may still be explained by a null vicariant model of allopatric divergence (Figure 2a). In this case, rather than being isolated by the northern plain states, *R. cingulata* and *R. indifferens* may instead have initially been separated between the SW and PNW into two large allopatric populations, with *R. cingulata* subsequently becoming subdivided across the SW, NoM, SoM, and ENA (Figure 2a). Such a modified vicariance hypothesis predicts that sharp genetic and phenotypic breaks should be observed associated with the geographic discontinuity between *R. cingulata* and *R. indifferens* in the SW versus PNW, respectively (Figure 2a). A degree of divergence may also potentially be observed among *R. cingulata* populations across the SW, NoM, SoM, and ENA, depending upon how long ago they became isolated. However, the arc of cherry‐infesting fly populations across the SW and NoM, as well as the existence of populations in SoM, also suggests the possibility of various alternative mixed modes of divergence as opposed to the null vicariance model. For example, populations in the SW and NoM could represent recent secondary contact between previously isolated ENA, PNW, and SoM populations (i.e., allo‐parapatry; Figure 2b). Alternatively, the current range of cherry flies could reflect a sequence of expansion from and fragmentation of what was once a continuous ancestral population across the SW and NoM (i.e., para‐allopatry; Figure 2c). Finally, more complex mixed modes are also possible in which the biogeography involves past allopatry of ENA, PNW, and SoM populations, followed by secondary contact across the SW and NoM, and then recent isolation (i.e., allo‐para‐allopatry; Figure 2d). If any of the alternate mixed‐mode models are true, then part of the history of speciation for cherry flies may predate their current isolation, with gene flow occurring during some stage(s) of their divergence. In contrast to the sharp geographic breaks expected due to vicariance (Figure 2a), gene flow accompanying the mixed‐mode scenarios would predict that cherry flies display clinal variation across the SW and NoM (Figure 2b–d). The existence of genetic and phenotypic clines would therefore suggest that flies in the SW and NoM once connected or served as the source of currently allopatric ENA, PNW, and SoM populations. Tentative support for clines comes from a morphological wing spot trait (Figure 3a) that varies from being common in ENA *R. cingulata* to rare in *R. indifferens* from the PNW (Bush, 1966), and anecdotally intermediate in frequency though the SW and NoM (J. Rull, personal observation).

In the current study, we characterize cherry fly populations across North America for variation in nuclear‐encoded microsatellite loci, maternally inherited mitochondrial DNA (mtDNA), and the wing spot morphological trait. Our primary objective is to distinguish between vicariance and a mixed mode of divergence by determining whether clinal variation and a pattern of IDB exist for current sky island isolates of cherry flies across the SW and NoM connecting *R. cingulata* in ENA with *R. indifferens* in the PNW, as well as potentially with *R. cingulata* in SoM. A secondary objective, given the existence of clines, is to evaluate whether the genetic data provide any insight into which of the various mixed‐mode scenarios may be occurring. In this regard, much is known about the history of climate change and its effects on the biogeography of the flora and fauna of the SW and NoM (Dansgaard et al., 1993; Martin & Harrell, 1957; McDonald, 1993). Specifically, this region is known to have undergone a series of dramatic climatic oscillations during the Pleistocene driven by major ice ages occurring at ~100,000 year‐intervals (Hewitt, 1996). These ice ages were punctuated by interglacial periods, the latest one beginning ~11,700 years ago (ya), demarcating the start of the Holocene (Hewitt, 1996). During the cooler and wetter ice ages, forests and woodlands, and their associated flora and fauna, were more contiguous across the SW and NoM (Betancourt, Van Devender, & Martin, 1990), fostering migration and gene flow. In contrast, during the intervening interglacial periods, warmer and drier conditions drove the forests and woodlands to higher elevation sky islands separated by low‐lying deserts, restricting gene flow.

The information on climate change can be used in conjunction with the current distribution of cherry flies to generate predictions concerning expected divergence times of populations under the differing mixed‐mode hypotheses. As cherries, and hence cherry flies, would be associated with the changing distributions of forest and woodland habitats, ice ages during the Pleistocene would represent times when fly populations were more contiguous through the SW and NoM. Thus, during glacial periods, gene flow would be possible, fostering the potential formation of clines. In contrast, interglacial periods, when cherries and cherry flies were restricted to isolated pockets in sky islands, as is currently the case, would correspond to times of limited or no gene flow. Consequently, during interglacial periods, populations could diverge in allopatry. Given that the most recent warming and drying started 8,000 to 9,000 ya in the early Holocene (Allen, Betancourt, & Swetnam, 1998), if the allo‐parapatry hypothesis is true, then this would imply recent allopatric separation of ENA, PNW, and SoM populations with potentially human‐mediated secondary contact in more recent historic times in the SW and NoM (Figure 2b). Under the para‐allopatry hypothesis (Figure 2c), clinal variation across SW and NoM sky island populations would be primary and formed prior the early Holocene, possibly during to the preceding Wisconsin glaciation 11,700 to 110,000 ya (Walker et al., 2009), when gene flow through the region was potentially high. Subsequently, warming and drying during the Holocene resulted in the isolation of cherry fly populations across North America. Finally, the allo‐para‐allopatry hypothesis (Figure 2d) would imply that geographically isolated ENA, PNW, and SoM populations likely existed in the interglacial period from 110,000 to 150,000 ya prior to the Wisconsin glaciation. Then, during the Wisconsin glaciation, cherry flies came into contact from these regions and formed secondary clines. Subsequently, warming and drying during the Holocene fragmented cherry flies into the present day isolated subpopulations currently distributed across North America.

## 2 MATERIALS AND METHODS

### 2.1 Study sites and specimens

All flies were directly collected as larvae in infested cherry fruit from the field and reared to adulthood in the laboratory using standard *Rhagoletis* husbandry methods (Feder, Chilcote, & Bush, 1989) before being frozen at −80°C for later genetic analysis. Only one egg is typically deposited per cherry, with a maximum of one larva emerging per each infested fruit (Bush, 1966). Thus, there is not an issue with having to choose to genotype one individual per cherry to avoid possible problems in biasing estimates of gene or haplotype frequencies due to scoring of related individuals. Flies from 17 populations sampled between 2004 and 2011 were genotyped for microsatellites and/or mtDNA (Figure 1a; Table S1). Previous surveys of *R. cingulata* in Mexico indicated that while the host black cherry is contiguous in the SMOr, the fly is now found only in two northern and southern areas of this mountain range (Rull et al., 2011), both sampled here (Figure 1a). Similarly, while *P. serotina* is present in the Sierra Madre Occidental Mountains of Mexico, flies have not been found in the area (Rull et al., 2011). Bitter and black cherries co‐occur in areas of the states of Arizona and New Mexico (Figure 1a). However, only black cherries were sampled from the SW, as bitter cherry is rare. The presence of both black and bitter cherries in the SW suggests that these two host plants and their associated fly populations cooccurred in the region in the past. *Rhagoletis cingulata* and *R. indifferens* are known to use other cherry species in common as secondary hosts (Bush, 1966). Thus, host affiliation is not likely to be a strong barrier to gene flow. In the absence of nonhost related pre‐ or postzygotic RI, if these two taxa overlapped in the past, or were to cooccur in the future, then they could potentially hybridize, thereby generating clines. In this regard, mating trials between *R. cingulata* from ENA and *R. indifferens* from the PNW showed moderate prezygotic isolation (isolation index = 0.27 = the percentage of intraspecific subtract interspecific matings in trials divided by the total percentage of matings; Hood, Egan, & Feder, 2012). In addition, Doellman et al. (2019) reported no evidence for strong postzygotic isolation in crosses between PNW and ENA cherry flies, further supporting the likelihood that *R. cingulata* from ENA and *R. indifferens* from the PNW would form a hybrid zone and even possibly fuse if they were to come back into contact.

### 2.2 Multilocus nuclear data

A total of 364 adult flies from populations 1–15 (Figure 1a; Table S1) were genotyped for 21 microsatellite loci (see Supplemental Methods for details). References for primers and conditions used for PCR amplification can be found in Maxwell, Thistlewood, and Keyghobadi (2013) and Michel et al. (2010). Due to their relatively high rates of mutation and levels of polymorphism (Estoup & Angers, 1998), we anticipated that the microsatellites should be sensitive indicators of demographic change, and informative for detecting clines and patterns of IBD among cherry fly populations. Genotyping was performed as described in Saint Jean et al. (2018), with size standards included in each genotyper run, as well as representative eastern *R. cingulata* and western *R. indifferens*, to ensure that alleles were properly aligned and comparably scored. Details of the methods used for microsatellite data analysis are given in the Supplemental Methods. We first constructed a genetic distance network useful to graphically summarize the genetic relationships among cherry fly populations across North America and visualize possible patterns of IBD using powermarker v3.25 (Liu & Mus, 2005). This was followed by Mantel tests of IBD using vegan (R package version 2.2‐1; Oksanen et al., 2015). We also quantified levels of genetic divergence within and between regions by calculating Jost's *D* values (*D*_Jost_) with the R package diversity, as suggested for highly polymorphic microsatellite loci (Jost, 2008; Keenan, McGinnity, Cross, Crozier, & Prodöhl, 2013). Tests for evidence of genetic subdivision among fly populations were next performed using structure v2.3.4 (Evanno, Regnaut, & Goudet, 2005; Pritchard, Stephens, & Donnelly, 2000). Lastly, we estimated divergence times between populations based on the Metropolis‐coupled Markov Chain Monte Carlo (MCMC) sampling algorithm in ima2p (Sethuraman & Hey, 2016). We focus on reporting results assuming an average microsatellite mutation rate of 6.3 × 10^–6^ per meiosis, which generated divergence time estimates for cherry flies corresponding well to known climatic changes in Holocene (see Sections 3 and 4).

### 2.3 Mitochondrial DNA

Mitochondrial DNA sequence data were generated for a total of 97 flies for a 1,482 bp fragment that included parts of the cytochrome oxidase I (COI), leucine transfer RNA (tRNA‐Leu), and cytochrome oxidase II (COII) genes (Table S1; GenBank accession numbers: KT221476–KT22179), using the overlapping primer pairs UEA5 and UEA10 (Lunt, Zhang, Szymura, & Hewitt, 1996) and C2‐J‐3138 and TK‐N‐3782 (Simon et al., 1994). Due to its lack of recombination and maternal inheritance, we anticipated that analysis of mtDNA haplotypes could provide useful information to detect and date past periods of geographic isolation for cherry flies. Methods for Sanger sequencing of mtDNA are described in Feder et al. (2003). Sequences were edited manually and assembled using codoncode aligner v3.7 (Codon Code Corp). A haplotype network was constructed for the mtDNA data using pegas (R package version 0.11; Paradis, 2010).

### 2.4 Morphological analysis

Morphological analyses were performed for four cherry fly populations collected in 2017–2018: *R. indifferens* from the PNW (site 4), *R. cingulata* from the SW (sites 7 and 8), and *R. cingulata* from ENA (site 15). These four sites were chosen (a) to reflect a representative sample of populations from eastern to western North America; and (b) because large sample sizes of flies with undamaged wings following freezing allowed for the frequency of the apical spot to be accurately estimated. Unfortunately, we inadvertently processed many flies from sites in the SW and Mexico for genetic analysis before mounting their wings for phenotypic analysis, restricting more detailed characterization of wing spot variation across the region to a future time when additional samples can be collected. Adult flies were scored by visual inspection under a 20× light microscope for the presence of the wing spot (Figure 3a). A chi‐square test was used to compare population proportions of flies with wing spots (Zar, 1999).

## 3 RESULTS

### 3.1 Clinal patterns in multilocus nuclear data

The genetic distance network for microsatellites shows a pattern of IBD in extant cherry fly populations across North America (Figure 1b). Populations of *R. cingulata* and *R. indifferens* were arrayed in the network in relation to their geographic location from ENA to Mexico (north to south), to the SW, to the PNW. As a result, a significant correlation was observed between geographic and overall genetic distance (IBD) for all 21 microsatellite loci across the 15 populations genotyped in North America (*r* = .81; *p* < .0001, as determined by nonparametric Monte Carlo simulations; Table 1). Significant genetic IBD was not only observed for the 21 microsatellites analyzed together but also for 19 of the 21 loci (90.5%) considered individually (Table 2). Although microsatellite genetic distance between populations was strongly correlated with geographic distance overall, the pattern was largely driven by sites in the SW and Mexico (Table 1; Figure 1b). Thus, among the six SW and Mexican populations of *R. cingulata*, significant IBD was observed (*r* = .86; *p* < .0001; Table 1). However, significant IBD was not detected among the six *R. indifferens* populations in the PNW (*r* = .48, *p* = .071) or among the three *R. cingulata* populations in ENA (*r* = −.22, *p* = .50).

**TABLE 1:** Correlation coefficients (*r*) and significance levels (*p*‐value) for Mantel tests of Isolation by distance (IBD) for the combined 21 microsatellite dataset scored across cherry‐infesting fly populations spanning indicated geographic regions (North America = sites 1–15; Pacific Northwest = sites 1–6; southwest USA & Mexico = sites 7–12; eastern USA = sites 13–15)

Isolation by distance test	*r*	*p*‐Value	Distance (km)
North America	.81	<.0001	4,000
Pacific Northwest	.48	.071	700
Southwest USA and Mexico	.86	<.0001	1,800
Eastern USA	−.22	.50	1,300

Note

Also given is the Euclidean distance (in km) separating the most distant sites in each region.

**TABLE 2:** Mantel tests for Isolation by distance (IBD) for individual microsatellite loci scored across North America (sites 1–15)

Locus	*r*	*p*‐Value
WCFF007	.27	**
WCFF024	.22	***
WCFF031	.46	****
WCFF057	.58	****
WCFF061B	.68	****
WCFF067	.44	****
WCFF083	.64	****
WCFF084A	.70	****
WCFF086A	.54	****
WCFF093	.35	***
WCFF105	.63	****
WCFF111	.36	***
P4	.48	****
P27	.58	****
P36	.13	.087
P37	.46	****
P45	.09	.1842
P50	.21	*
P54	.29	**
P71	.22	**
P80	.28	**

* *p* < .05.

** *p* < .01.

*** *p* < .001.

**** *p* < .0001.

Estimates of *D*_Jost_ within and among regions displayed patterns of differentiation matching those seen in the IBD analysis (see Table S2 for mean pairwise *D*_Jost_ values for all 21 loci between populations). For example, the average *D*_Jost_ value for all 21 loci among *R. indifferens* populations in the PNW was 0.0085 ± 0.0015*SE* (*n* = 15 pairwise comparisons), while it was 0.0086 ± 0.0045*SE* (*n* = 3) among *R. cingulata* populations in ENA. In contrast, the mean *D*_Jost_ between *R. indifferens* populations in PNW and *R. cingulata* populations in the ENA was 0.3232 ± 0.0077*SE* (*n* = 18), while it was 0.1369 between the *R. cingulata* population at site 7 in Arizona in the SW and site 11 in NoM.

Clinal variation was also observed when, instead of geographic and overall genetic distance, longitude was plotted against the mean population frequency of microsatellite alleles more common in ENA than the PNW (Figure 3b). No distinct break in allele frequencies was therefore apparent between cherry‐infesting fly populations in North America, at least involving ENA, SW, NoM, and PNW populations. Indeed, in all cases, where an allele was segregating at a mean frequency of >0.05 in *R. indifferens* in the PNW and absent in ENA, the variant was present in at least one population, and often several or all of the sites, in the SW and Mexico, while the reverse was true for alleles found in at a mean frequency of >0.10 in the ENA and absent in the PNW (Table S3). The situation differed slightly for the SoM sites, as two microsatellites (WCFF24 and WCFF105) possessed unique alleles in these two populations at combined high frequencies not found elsewhere (Table S3). In addition, the two SoM populations had more alleles unique to the region not found at any other site (*n* = 45) than both the three ENA populations (*n* = 25) and six PNW populations (*n* = 21) surveyed (Table S3).

### 3.2 Population structure from multilocus nuclear data

structure analysis implied that the clines were due to fly populations differing in the frequencies of alleles they possess rather than representing differences in the proportions of parental *R. indifferens* and *R. cingulata* genotypes across sites (Figure 3c). The largest change in log likelihood in the structure analysis was observed around *K* = 2 (Table 3), implying the existence of two diverged cherry‐infesting fly populations, representing *R. indifferens* in the PNW and *R. cingulata* in ENA connected by a series of admixed populations through the SW and NoM. Although seven individuals at Fort Davis, Texas (site 8) did have multilocus genotypes representative of *R. indifferens* in the PNW or *R. cingulata* in ENA, the remaining 16 flies (~70%) had admixed genotypes (Figure 3c). Moreover, in no other population in the SW and NoM were flies of both parental genotypes detected.

**TABLE 3:** Mean estimated *Ln* likelihood, standard deviation, and ∆*K* (Evanno et al., 2005) calculated across 10 replicate structure analyses including all 15 cherry‐infesting fly populations for *K* = 1–15, using 500,000 burn‐in iterations followed by 1,000,000 MCMC repetitions under the correlated allele frequencies with admixture model

*K*	*Ln Lik*	*σ*	Δ*K*
1	−20,092.3	0.11	−
2	−16,957.42	0.59	4,262.25
3	−16,346.52	244.92	0.80
4	−15,539.64	1.01	671.13
5	−15,408.5	308.55	0.91
6	−14,996.27	49.53	5.48
7	−14,855.54	60.34	0.01
8	−14,715.41	30.15	1.02
9	−14,606.08	3.59	46.32
10	−14,663.07	39.17	1.43
11	−14,664.08	53.99	3.12
12	−14,833.35	77.48	0.25
13	−14,983.36	192.77	0.69
14	−14,999.85	303.14	1.33
15	−15,420.87	256.32	3.68

Note

The gray row indicates the best fit number of subpopulations for *K* = 2.

### 3.3 Divergence estimates from multilocus nuclear data

The estimated divergence time with the highest posterior probability between *R. indifferens* in the PNW (site 4) and *R. cingulata* in Arizona (site 7), assuming a mutation rate of 6.3 × 10^–6^, was 5,556 ya (95% credible interval 2,381 to 14,762 ya). Similarly, populations of *R. cingulata* in NoM (site 11) and in ENA (site 15) were estimated to have diverged 8,413 ya (95% credible interval 3,651 to 20,476 ya). Among the SW and NoM populations, *R. cingulata* in Arizona (site 7) and western Texas (site 8) were estimated to have separated from each other 4,921 ya (95% credible interval 2,063 to 12,540 ya), while western Texas and Nuevo León (site 11) diverged 2,381 ya (95% credible interval 794 to 7,460 ya). In comparison, the separation of SoM (site 9) and NoM (site 11) Mexican populations of *R. cingulata* dated to 16,032 ya (95% credible interval 9,048 to 32,857 ya), comparable to the estimate of 15,079 ya (95% credible interval 7,143 to 31,270 years) between *R. indifferens* in the PNW (site 4) and *R. cingulata* in ENA (site 15). We note that divergence time estimates among cherry fly populations depend on the mutation rate assumed in the ima2p analysis. The results above were based on a rate of 6.3 × 10^–6^ that corresponds to that of *Drosophila* (Schug, Mackay, & Aquadro, 1997). However, using higher mutation rates in the analysis resulted in proportionately more recent estimates of population divergence (Figure S3).

### 3.4 Mitochondrial DNA divergence

**FIGURE 4:**
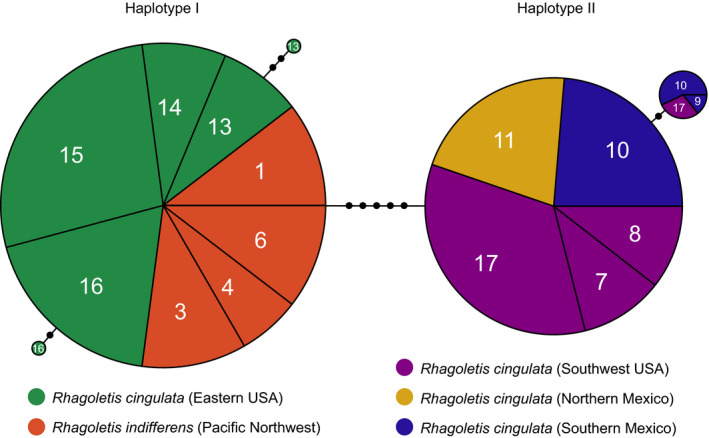
Haplotype network for mitochondrial DNA (mtDNA) derived from 1,482 total bp of COI, tRNA‐Leu & COII, sequenced for cherry‐infesting *Rhagoletis* across North America. The sizes of the circles are proportional to the number of identical haplotypes sequenced from different individuals (from 2 to 48), with site number designated as in Table S1 and Figure 1a and geographic region denoted by color. Small black circles represent the number of base‐pair substitutions differentiating sampled haplotypes. Note that in contrast to microsatellites, *R. indifferens* in the Pacific Northwest (PNW) and *R. cingulata* in the eastern USA (ENA) share the same major haplotype in common (haplotype I), while *R. cingulata* from the southwest USA (SW) and Mexico possess a different major haplotype (haplotype II)

Mitochondrial DNA haplotypes displayed a different pattern of geographic variation compared to the microsatellites (Figure 4). In contrast to the microsatellites, the same major mtDNA haplotype was shared between *R. indifferens* in the PNW and *R. cingulata* in ENA (haplotype I). Minimal mtDNA variation was observed for haplotype I within both the PNW and ENA populations. A second major mtDNA haplotype II was observed for flies from the SW and Mexico that showed a minimum of five bp substitutions (~0.34% sequence divergence) from the PNW and ENA haplotype. Again, there was little mtDNA variation detected within the SW and Mexico for haplotype II; one variant was found in seven individuals across New Mexico (site 17), San Martín Texmelucan, Mexico (site 9), and Huamantla, Mexico (site 10) that differed by one bp substitution from the other 38 flies sequenced. Thus, rather than displaying clinal variation and IBD similar to microsatellites, mtDNA showed a disjunct break in the middle of the range of cherry flies in the SW and Mexico. Assuming a mtDNA molecular clock for insects of 3.4% pairwise divergence between taxa per million years (Papadopoulou, Anastasiou, & Vogler, 2010), the five bp difference seen between the SW/Mexican haplotype II and the PNW/ENA haplotype I would translate into a coalescence time of ~100,000 years. The estimate is ~157,000 years given a clock of 2.3% divergence per million years (Brower, 1994).

### 3.5 Clinal patterns in morphological variation

The proportion of flies with apical wing spots varied significantly across geography (*X* ^2 ^= 572.36; *df* = 4; *p* < .0001), in a manner similar to the pattern for microsatellites (Figure 3). A high proportion of *R. cingulata* at site 15 in ENA had a wing spot (80%, *n* = 350) compared with *R. indifferens* at sites 4 and 5 in the PNW (13%, *n* = 332 and 15%, *n* = 558, respectively; both sites significantly differed from site 15, with *p* < .05). Flies in the SW had intermediate wing spot frequencies (site 7 = 60%, *n* = 106; site 8 = 61%, *n* = 456; both sites significantly differed from sites 4, 5, and 15, *p* < .05 in all cases).

## 4 DISCUSSION

### 4.1 Null vicariant versus mixed‐mode divergence

With respect to our primary objective, the results for the microsatellites reveal a clinal pattern of allele frequency differences in cherry fly populations across the PNW, SW, ENA, and NoM (Figures 1b and 3b,c), as predicted for a mixed mode of divergence. Populations from SoM also fit the general IBD pattern for microsatellites (Figure 1b). However, there appears to be a geographic gap in the distribution of extant fly populations between northern and southern Mexico, although black cherry hosts are present (Rull et al., 2011). Thus, we have no genetic information as to whether populations connecting SoM and NoM sites in the past displayed clinal variation for microsatellites. However, SoM populations, like those in NoM and the SW, tend to possess microsatellite alleles found in ENA and not the PNW (see green highlighted boxes in Table S3) and vice versa (see orange boxes in Table S3). The pattern of shared variation suggests that if connecting sites existed or may be discovered bridging northern and southern Mexico, they would be intermediate in microsatellite frequencies, reflecting a clinal pattern of variation between the two regions. The wing spot phenotypic trait also differed across the four United States populations measured in a manner consistent with clinal variation, as the two sites in the SW in Texas and Arizona are intermediate in frequency between those in ENA and the PNW (Figure 3a). However, more extensive analysis of populations throughout the SW and Mexico is needed to determine how fine scale the clinal pattern of variation is across sites. Regardless, the general results for nuclear‐encoded genetic and morphological wing spot variation are supportive of IBD among cherry fly populations across North America. Thus, the recent history of cherry flies does not appear to involve a simple allopatric bifurcation, as no clear microsatellite or phenotypic break exists between *R. indifferens* populations in the PNW and *R. cingulata* in the SW and NoM (see below for discussion of the different pattern for mtDNA). The finding of clinal variation therefore implies that the history of cherry flies involves some form of mixed‐mode divergence.

### 4.2 Assessing different mixed‐mode models

Given the finding of clinal variation, our secondary objective was to assess whether patterns of genetic differentiation and estimated divergence times for populations, when coupled with the known climatic history of the SW and NoM, might provide insight into the likelihoods of different mixed modes of speciation for cherry flies. As discussed in the introduction, there are three major possibilities (Figure 2b–d). We discuss below why the first allo‐parapatric hypothesis (Figure 2b), involving secondary contact during the Holocene, is unlikely given the current geographic isolation of flies across North America. Instead, we suggest that our results best conform to para‐allopatric hypothesis two (Figure 2c), at least with respect to those populations in the PNW and ENA. However, the presence of the diverged mtDNA haplotype II through the SW and Mexico does not rule out hypothesis three of allo‐para‐allopatric divergence (Figure 2d), specifically in regard to the relationship of SoM flies with those in the SW and NoM. We thus suggest that the contrasting patterns of differentiation between microsatellites and mtDNA may reflect a multiple mixed mode of divergence for cherry flies across the SW and NoM, representing a composite of both primary IBD across the region together with secondary contact involving populations from SoM. While conjectural, we elaborate below on how such a scenario could account for certain aspects of the distribution of microsatellite alleles across North America, as well as for the presence of diverged mtDNA haplotype II in the SW and NoM shared with SoM populations that appears interjected between ENA and PNW populations possessing an alternate mtDNA haplotype I in common.

### 4.3 Hypothesis one: allo‐parapatric model

Hypothesis one contends that the microsatellite clines observed across the SW and NoM were formed by recent secondary contact during the Holocene of isolated fly populations from the PNW, ENA, and SoM that diverged previously in allopatry (Figure 2b). We consider this scenario unlikely for several reasons. First, given the current physical separation of cherry flies on sky islands across the SW and NoM, there is little likelihood of migration and gene flow among these populations at the present time. Second, the current isolation of cherry flies corresponds climatically and dates genetically to the Holocene, when warmer and drier conditions starting 8,000 to 9,000 ya forced forest and woodland associated plants and animals to higher elevations (Allen et al., 1998). We assumed a microsatellite mutation rate of 6.3 × 10^–6^ for our estimates of divergence time for cherry flies, which corresponds to that of *Drosophila* (Schug et al., 1997) and represents the lower end of rates for insects (Estoup & Angers, 1998). Thus, divergence time is not likely to be much greater than those we report, as this would require unrealistically low mutations rate. However, it is possible that actual mutation rates in cherry flies are higher than 6.3 × 10^–6^, which would proportionately reduce estimated divergence times. For example, a mutation rate of 1 × 10^–3^ would change the estimated divergence time between *R. indifferens* in the PNW and *R. cingulata* in Arizona from 5,556 to 50 ya (Figure S3). Such a timeline would seem to make the hypothesis of recent secondary contact more plausible. However, such recent divergence times would require that cherry flies were independently introduced into the SW and NoM from allopatric PNW, ENA, and SoM populations within historical times, and there is no record of this (Bush, 1966). Also, following these introductions, cherry flies from the different regions would have subsequently had to spread rapidly in an island‐hopping fashion among mountain isolates to generate the observed microsatellite clines, which seems highly unlikely. Third, and finally, a scenario of recent introductions would have difficulty in accounting for the lack of mtDNA variation throughout the SW and Mexico. Given recent gene flow from the PNW, ENA, and SoM posited by hypothesis one, we would expect to observe polymorphism for the two major mtDNA haplotypes I and II across the SW and NoM, which is not the case, as only haplotype II is present.

### 4.4 Hypothesis two: para‐allopatric model

Setting aside consideration of the diverged mtDNA haplotype II in the SW and Mexico, hypothesis two (para‐allopatric model) appears to best explain the pattern of microsatellite and wing spot variation observed across North America, particularly with respect to the PNW and ENA (Figure 2c). First, the estimated divergence time of 15,079 ya between *R. indifferens* in the PNW and *R. cingulata* in ENA corresponds well with when ice sheets were retreating after the last glacial maximum, which would have allowed the ancestral population across the SW and NoM to expand northward into these two regions. Second, the estimated divergence times from 5,556 ya to 8,413 ya between flies in the PNW and ENA versus those in the SW and NoM, respectively, matches the timeline for when climate change in the early Holocene 8,000–9,000 ya would have started to isolate cherry fly populations across North America. Third, levels of microsatellite variation among populations within the PNW and ENA are reduced, with no significant IBD observed within these two regions, compared to variation across the SW and NoM (Table 1), as might be expected if cherry flies in the PNW and ENA represent recent range expansions from a more diverse ancestral population. Fourth, flies in the PNW and ENA share the same mtDNA haplotype I in common and there is limited mtDNA variation within either region (Figure 4), which again implies a recent origin and lack of previous allopatry between flies in these two regions. It may seem odd that mtDNA haplotype I is identical between the ENA and the PNW given their estimated divergence time of 15,079 ya based on the microsatellites. However, assuming a mtDNA molecular clock of 3.4% pairwise difference between taxa per million years and not considering the coalescent time within the ancestral population, observing no fixed substitution difference for the 1,482 bp region of mtDNA haplotype I we sequenced would be predicted to be observed ~47% of the time in 15,079 years (=0.99949^1,482^). There is a major difficulty with the para‐allopatric hypothesis, however, which concerns the presence of the diverged mtDNA haplotype II in the SW and Mexico. As alluded to above and discussed below, we hypothesize that the mtDNA discrepancy may reflect a cycle of allopatry and secondary contact involving flies from SoM dating to the interglacial period 100,000 to 150,000 ya preceding the Wisconsin glaciation.

### 4.5 Hypothesis three: allo‐para‐allopatric model

We argue that hypothesis three (Figure 2d) involving an allo‐para‐allopatric sequence of events is not the most parsimonious explanation for the pattern of differentiation in the PNW and ENA but that a variation of the scenario may help account for the diverged mtDNA haplotype II observed through the SW and Mexico. With respect to the PNW and ENA, there is no genetic evidence for these populations having been allopatrically isolated during the interglacial period 100,000–150,000 ya prior to the Wisconsin glaciation. The estimated divergence time between PNW and ENA based on the microsatellites is 15,079 ya (95% credible interval 7,143 to 31,270 years), which would place any past allopatry, if it occurred, in the Wisconsin glaciation.

One possible scenario that could account for allopatry during the Wisconsin glaciation is that populations in the PNW and ENA resided in one or more northern refugia during this time. Thus, instead of expanding northward following retreating ice sheets, current PNW and ENA populations migrated southward from refugia as conditions warmed to contact one another and form clines across the SW and NoM by the early Holocene. While seemingly plausible, the refugium/refugia scenario of secondary contact has several problems. For example, if two refugia were involved (possible candidates being the west coast of Alaska/British Columbia and off the east coast of New England), then it would be difficult to explain how current populations in the PNW and ENA that emanated from these refugia share the same mtDNA haplotype I in common. A potential solution to this issue may be that PNW and ENA were actually connected at some time by a conduit through the North, permitting gene flow and, thus, the homogenization of mtDNA haplotypes between the two regions. In this regard, the pin cherry, *Prunus pensylvanica*, currently has a distribution through Canada connecting bitter cherry, the main host of *R. indifferens* in the PNW, with black cherry, the major host of *R. cingulata* in ENA (Figure S4). Maxwell et al. (2013) have reported rearing *R. indifferens* from pin cherry fruit collected in British Columbia, Canada. Pin cherry has also been cited as an alternate, although rare, host for *R. cingulata* in ENA (Bush, 1966). If true, then cherry flies may actually have formed a ring around North America at some time in the past (Irwin, Irwin, & Price, 2001). However, pin cherry is also the primary native host for *R. fausta*, a fly that outcompetes *R. cingulata* on *P. pensylvanica* (Bush, 1966). Moreover, our attempts at rearing *R. cingulata* from pin cherries in the states of Wisconsin and Minnesota, USA have to date been unsuccessful. Thus, there are reasons to doubt that pin cherry is or was an effective bridge between PNW and ENA populations of cherry flies now or in the past.

A single refugium scenario also has difficulties. For example, given that it does not appear likely for pin cherry to have served as a conduit for east/west movement of flies, some alternative and not readily apparent means would need to be discerned for how migrants spread from a hypothesized single refugium to subsequently come into secondary contact in the SW and NoM. In addition, while the existence of a single refugium could explain the lack of mtDNA haplotype I divergence between the PNW and ENA, it would also predict that a genetic signature should be observed for microsatellites reflecting the period of isolation for these flies in the refugium. However, this is not the case for the microsatellites, as there are no alleles of consequential frequency shared uniquely among all fly populations in the PNW and ENA and absent from the SW and Mexico (Table S3).

While the current data do not provide evidence for glacial refugia/refugium in the North and are most consistent with a primary origin for the microsatellite clines connecting PNW and ENA populations, this may not be the case for the diverged mtDNA haplotype II found in the SW, NoM, and SoM. In this regard, the estimated divergence time between mtDNA haplotypes I and II is 100,000–150,000 ya, depending upon the rate considered for the molecular clock. This timeline corresponds with the interglacial period preceding the Wisconsin glaciation when, similar to the current time, cherry fly populations in SoM may have been isolated from those elsewhere. Thus, it is conceivable that the diverged mtDNA haplotype II may reflect a period of past allopatry for cherry flies. In accord with this scenario, flies from SoM do possess a greater number of microsatellite alleles unique to the region (*n* = 45) than are found in the PNW (*n* = 21) and ENA, despite fewer numbers of sites and flies being genotyped from SoM (see blue boxes in Table S3). Indeed, for the five loci WCFF24, WCFF93, WCFF105, WCFF11, and P27, the alleles unique to SoM populations are either individually or collectively present at appreciable frequencies >0.10. Thus, there is better evidence for a nuclear genetic signature of past allopatry for the SoM compared with PNW and ENA populations.

The allo‐parapatric scenario for SoM flies, if true, still must explain certain anomalies in the data. Most importantly, if secondary contact and gene flow occurred between SoM, SW, and NoM flies, then why is mtDNA haplotype II fixed, while microsatellites display IBD among these populations (Tables 1 and 2)? Several factors alone or in combination may potentially account for differences in the apparent introgression dynamics between mtDNA and microsatellites, but still require testing to verify. These include differential lineage sorting related to the smaller effective population size for mtDNA (Charlesworth, Bartolome, & Noel, 2005), sex‐related differences in migration rates, the selective sweep of a favorable mtDNA variant (Galtier, Nabholz, Giémin, & Hurst, 2009; Silva, Lima, Martel, & Castilho, 2014), or the presence of a cytoplasmically inherited endosymbiont (e.g., *Wolbachia*; Brucker & Bordenstein, 2012; Jaenike, Dyer, Cornish, & Minhas, 2006; Werren, 1998). We return to the possibility of an endosymbiont below when we discuss what is currently known concerning patterns of RI among cherry flies across North America.

In summary, it appears that a combination of hypotheses two and three may best explain the history of cherry flies in North America based on the current genetic and phenotypic data. Thus, while the presence of clines was confirmed in the present study, the disjunct pattern for mtDNA complicates the story. Additional DNA sequencing studies could help to further resolve and complement the microsatellite and mtDNA results. In particular, sequences for nuclear‐encoded genomic regions experiencing low rates of recombination may confirm or discount past allopatry. In other *Rhagoletis* flies showing evidence of mixed geographic modes of speciation (Xie et al., 2007), sequencing of inversion polymorphisms has proven useful for inferring secondary contact when rearrangements mapping to different chromosomes were shown to have similar divergence times (Feder et al., 2003, 2005). It is not known if *R. indifferens* and *R. cingulata* possess shared inversion polymorphisms but, if so, then a similar strategy may be applied to cherry flies to test for evidence of secondary contact. In this case, inversions originating in a time of past allopatry would capture and tend to retain and accumulate differences dating to the time of the original geographic isolation of populations plus the coalescence times of variants within the ancestral population, which should be similar among loci. Thus, for example, if we find multiple different rearrangements in cherry flies in the SW and Mexico that have estimated divergence times roughly matching mtDNA haplotype II in the range of 100,000–150,000 ya, then this would provide additional support for hypothesis three. Moreover, if such a pattern is not found elsewhere and divergence times for all colinear regions of the genome correspond to those estimated for the microsatellites, then this would strengthen the case for hypothesis two for PNW and ENA flies. It would also be informative to determine if and how the genetics and biogeography of cherry flies match that of their bitter and black cherry hosts. Given the reliance of flies on infesting the fruit of their host plants, finding similar patterns between cherries and cherry flies would strengthen inferences concerning modes of divergence for the flies. In this regard, Guzmán, Segurab, Aradhyac, and Potter (2018) reported microsatellite differentiation among *P. serotina* in Mexico and central Texas. However, their sampling scheme did not match ours to allow testing for geographic concordance between flies and black cherries. Future studies of the genetics of cherry hosts at collecting sites paired with flies would therefore provide another potentially fruitful means to help further resolve different mixed‐mode hypotheses for the divergence of cherry flies.

### 4.6 Evolution of RI

As outlined in the Introduction, the problem of verifying a mixed mode of speciation is two‐fold. First, one must characterize the biogeography of a pair of taxa to demonstrate that it has changed through time. Second, one must show that during times when taxa were both in contact and experiencing gene flow, and geographically separated and isolated, they evolved RI. We have accomplished the first goal in the current study. The genetic and phenotypic patterns of clinal variation found for cherry flies imply that they have a biogeography consistent with a mixed mode of speciation and our results shed light on which of the various types of mixed‐mode models appear most likely to be responsible for generating the observed patterns. However, our findings do not directly address the second issue of the evolution of RI. Thus, we may be able to infer that genetic differences likely accrued between cherry flies during periods of parapatry and allopatry but this does not demonstrate that RI also evolved at these times. Further work measuring levels of pre‐ and postzygotic RI are therefore needed to assess the second component of mixed‐mode speciation for cherry flies.

In regard to the question of RI, Hood et al. (2012) documented moderate levels of prezygotic isolation between *R. cingulata* from ENA and *R. indifferens* from the PNW (isolation index = 0.27). If additional crosses involving flies from the SW and NoM show intermediate levels of prezygotic isolation compared to that observed between flies from ENA and the PNW, then a case could be made for a degree of prezygotic RI evolving in parapatry, possibly related to the wing spot phenotype. Doellman et al. (2019) reported no evidence, however, for significant postzygotic isolation in crosses between PNW and ENA cherry flies. While strengthening the case that flies from these two regions are recently derived, the apparent lack of postzygotic isolation leaves unresolved the question of whether any RI in cherry flies has allopatric origins. However, Tadeo et al. (2015) reported significant reductions in fecundity when *R. cingulata* from Coahuila in northern Mexico (site 12) were crossed to *R. cingulata* from Kearneysville, West Virginia, in ENA (a site not genotyped here). Additional crosses are needed to characterize whether and how the postzygotic RI observed by Tadeo et al. (2015) relates to other regions, particularly with regard to the SW and PNW. Depending upon the results, it may be possible to infer that RI has also evolved in allopatry for cherry flies, in this case postzygotic.

It would be particularly intriguing if the pattern of postzygotic isolation reported by Tadeo et al. (2015) correlates with the disjunct distribution of mtDNA haplotypes I and II. Such a finding would be consistent with the possibility of a cytoplasmic basis for RI, perhaps involving a sweep of an endosymbiont like *Wolbachia*, as has been shown for other insects (Brucker & Bordenstein, 2012; Jaenike et al., 2006; Werren, 1998). While speculative, Schuler et al. (2013) reported the presence of *Wolbachia* in native populations of *R. cingulata* in the USA and in introduced populations of the species in Europe. In addition, different strains of *Wolbachia* have been associated with specific mtDNA haplotypes and the occurrence of cytoplasmic incompatibility between different races of the European cherry fruit fly, *R. cerasi* (Arthofer et al., 2009; Riegler & Stauffer, 2002). Thus, there is precedent for *Wolbachia* sweeping through *Rhagoletis* populations (Bakovic, Schebeck, Telschow, Stauffer, & Schuler, 2018; Schuler et al., 2013, 2016). With regard to the results of Tadeo et al. (2015), if *Wolbachia* is involved, then we would predict that at least two strains of the endosymbiont differentiate Mexican from ENA cherry flies in order to account for the apparent bidirectionality of postzygotic RI.

### 4.7 Generality to other systems

Hybrid zones are ubiquitous in nature and represent clines associated with reproductive isolation, being created and maintained by a balance between migration and selection against individuals of mixed ancestry (Barton & Hewitt, 1981, 1985, 1989). Many hybrid zones have been inferred to be the result of secondary contact, usually since the last glaciation (Barton & Hewitt, 1985). Most hybrid zones have been argued to constitute “tension zones,” with hybrids suffering reduced fitness due to inherent genomic incompatibilities not necessarily tied to local environmental conditions (Barton & Hewitt, 1985; Gavrilets, 1997). Such hybrid zones can be relatively narrow, with gene and phenotype frequencies tending to change concordantly with one another (Barton & Hewitt, 1981, 1985, 1989).

Our results for cherry flies appear to differ in several respects from most hybrid zones. Rather than being narrow, clinal variation for cherry flies is relatively broad across North America, especially through the SW and NoM. In addition, the clines are not all concordant. While the vast majority of microsatellites display a significant pattern of IBD, the exact pattern of clinal variation differs some among alleles and loci. For example, in some cases, an allele present in ENA and absent from the PNW is sometimes found as far west as Arizona, sometimes as far west as Texas, and sometimes only as far west as NoM (Table S3). Moreover, unlike most tension zones, Doellman et al. (2019) reported no evidence for pronounced postzygotic RI between populations (PNW vs. ENA) flanking the cline. However, Tadeo et al. (2015) found strong RI for populations in NoM, residing within the clines displayed by microsatellites.

Cherry flies therefore possess attributes and may have a biogeographic history that differs from most classic hybrid zone systems, possibly reflecting a primary rather than secondary origin. Do other flora and fauna distributed across the SW and NoM share these attributes with cherry flies? In this regard, many studies have documented postglacial fragmentation of woodlands through the SW and Mexico (Cuenca, Escalante, & Piñero, 2003; Delgado, Piñero, Chaos, Pérez‐Nasser, & Alvarez‐Buylla, 1999; Delgado et al., 2007; González‐Rodríguez, Bain, Golden, & Oyama, 2004; Jaramillo‐Correa, Beaulieu, Ledig, & Bousquet, 2006; Ledig, Jacob‐Cervantes, Hodgskiss, & Eguiluz‐Piedra, 1997) and its effects on the genetic structure of forest‐associated plant and animal populations (Avise & Walker, 1998; Hewitt, 1996; Jaramillo‐Correa et al., 2008; Lessa, Cook, & Patton, 2003), including for insects (Knowles, 2000, 2001; Lobo & Halffter, 2000; Smith & Farrell, 2005), spiders (Masta, 2007), scorpions (Bryson, Riddle, Graham, Smith, & Prendini, 2013), reptiles (Bryson, Murphy, Graham, Lathrop, & Lazcano, 2011; Bryson, Murphy, Lathrop, & Lazcano‐Villareal, 2011; Wiens et al., 2019), birds (Johnson & Cicero, 2004; Weir & Schluter, 2004), and mammals (Ceballos, Arroyo‐Cabrales, & Ponce, 2010). Interestingly, the timing of these effects generally predates, sometimes substantially, the most recent warming trend across the SW and Mexico beginning 8,000–9,000 ya in the early Holocene (see McCormack, Bowen, & Smith, 2008). However, some studies that included nuclear‐encoded genes have reported evidence for genetic structuring after the last glacial maximum that ended ~18,000–20,000 ya (Clark et al., 2009; Favé et al., 2015; Holycross & Douglas, 2007; McCormack et al., 2008). But this is not always the case. For example, Wiens et al. (2019) found divergence times for the lizard *Sceloporus jarrovii* among different mountain ranges in Arizona ranging from 0.5 to 4.5 mya based on 1,495 nuclear‐encoded RADseq loci.

With respect to the issue of clines, latitudinal variation has been detected within some SW and NoM species, which has been argued to have resulted from the movement of populations as glaciers advanced and retreated during the Pleistocene (e.g., Perez‐Alquicira, Weller, Domínguez, Molina‐Freaner, & Tsyusko, 2018; Potter, Hipkins, Mahalovich, & Means, 2015). In addition, a suture zone has been postulated to exist between southeastern Arizona and southwestern New Mexico in which hybrid zones between trees, birds, and mammals appear to be concentrated above background (Swenson & Howard, 2005). Hewitt (1996, 1999) hypothesized that suture zones in North America, including the southeastern Arizona‐southwestern New Mexico zone, are mainly found in north‐south mountain chains associated with climatic warming and the retreat of glaciers. During glacial retreat, eastern and western taxa in refugia may have moved though mountain passes, come into contact, and hybridized. The longitudinal variation evidenced by the southeastern Arizona‐southwestern New Mexico suture zone differs from that for cherry flies, however, in that isolation in the former case would be generated by glaciation, while the reverse would be true for cherry flies. Thus, while climate change undoubtedly has affected the genetic structure of populations in the SW and NoM, the timing and details of the processes involved may differ between systems. As a result, it remains to be determined how many other systems in the SW and NoM possess attributes akin to cherry flies that allow for testing for a mixed mode of divergence. Cherry flies may have a relatively more recent history in the SW and Mexico compared with most other systems such that population divergence is reflected in clinal rather than more discrete differences between sky islands.

## 5 CONCLUSION

While potentially common, our study highlights how challenging it can be to assess the mixed‐mode hypothesis. Thus, aside from reinforcement evolving following secondary contact, evidence for mixed‐mode speciation is generally lacking. Our findings for *R. indifferens* and *R. cingulata* are consistent with a mixed mode of divergence for these flies. However, further DNA sequencing studies and analyses of pre‐ and postzygotic isolation among cherry fly populations are needed to strengthen the biogeographic implications of the current microsatellite and mtDNA data and to show that in addition to population divergence, RI also accrued during periods of parapatry and allopatry. Moreover, our findings suggest that different mixed modes of divergence may apply to different parts of the cherry fly range, further complicating the problem. Studies of other systems are also clearly needed to extend the results for cherry flies more generally. However, many species may not possess attributes ideal for detecting a mixed mode of divergence despite its occurrence. For example, if climate change continues and causes the extinction of cherry fly populations through the SW and NoM in the near future, then things would change. Instead of detecting clinal variation and inferring a mixed mode of divergence, a future study would likely conclude that PNW, ENA, and SoM populations represent allopatric isolates undergoing vicariant speciation. Thus, the dissimilarities between cherry flies and other systems may reflect the particular snapshot in time that they happen to capture rather than any inherent differences. In this regard, suture zones in which populations have been hypothesized to have undergone repeated cycles of allopatry, secondary contact, and gene flow associated with glaciation potentially represent rich systems to extend the results for cherry flies more distantly in time. Studies of more recently diverged species may help reveal if signatures of parapatric divergence can be found prior to allopatry. Regardless, our results also underscore the importance of analyzing different types of genetic markers and genomes with differing demographic and evolutionary dynamics to accurately and fully resolve the biogeography of a system and its consequences for speciation. Moreover, the possibility of cytoplasmic sweeps, perhaps due to endosymbionts, urges caution about inferring biogeographic history based on the assumption that cytoplasmically inherited genomes like mtDNA represent passive, neutral markers of population structure. Thus, while categorizing speciation into different modes and assuming neutral gene flow dynamics be can be helpful for understanding population divergence, life may often produce additional complications.

## CONFLICT OF INTEREST

All authors declare no conflict of interest.

## AUTHOR CONTRIBUTION

**Meredith M. Doellman:** Conceptualization (equal); Data curation (equal); Formal analysis (equal); Investigation (equal); Methodology (equal); Project administration (equal); Visualization (equal); Writing‐original draft (equal); Writing‐review & editing (equal). **Gilbert Saint Jean:** Conceptualization (equal); Data curation (equal); Investigation (equal); Methodology (equal); Resources (equal); Writing‐original draft (equal); Writing‐review & editing (equal). **Scott P. Egan:** Conceptualization (equal); Formal analysis (equal); Funding acquisition (equal); Investigation (equal); Methodology (equal); Resources (equal); Writing‐review & editing (equal). **Thomas H. Q. Powell:** Data curation (equal); Formal analysis (equal); Investigation (equal); Methodology (equal); Project administration (equal); Resources (equal); Visualization (equal); Writing‐review & editing (equal). **Glen R. Hood:** Conceptualization (equal); Formal analysis (equal); Funding acquisition (equal); Investigation (equal); Resources (equal); Writing‐review & editing (equal). **Hannes Schuler:** Formal analysis (equal); Investigation (equal); Resources (equal); Writing‐review & editing (equal). **Daniel J. Bruzzese:** Investigation (equal); Resources (equal); Visualization (equal); Writing‐review & editing (equal). **Mary M. Glover:** Formal analysis (equal); Resources (equal); Visualization (equal); Writing‐review & editing (equal). **James J. Smith:** Resources (equal); Writing‐review & editing (equal). **Wee L. Yee:** Formal analysis (equal); Funding acquisition (equal); Resources (equal); Writing‐review & editing (equal). **Robert Goughnour:** Resources (equal); Writing‐review & editing (equal). **Juan Rull:** Resources (equal); Writing‐review & editing (equal). **Martin Aluja:** Resources (equal); Writing‐review & editing (equal). **Jeffrey L. Feder:** Conceptualization (equal); Formal analysis (equal); Funding acquisition (equal); Investigation (equal); Resources (equal); Writing‐original draft (equal); Writing‐review & editing (equal).

## Supporting information

Supplementary Material

Table S3

## Data Availability

GenBank accession numbers for mtDNA sequences: KT221476–KT22179. Microsatellite genotypes for all individuals have been deposited in DRYAD (https://doi.org/10.5061/dryad.47d7wm3b6).
