# Supplementary material for: Evidence for spatial clines and mixed geographic modes of speciation for North American cherry‐infesting Rhagoletis (Diptera: Tephritidae) flies
Source: Ecol Evol. 2020 Oct 28;10(23):12727–44. doi: 10.1002/ece3.6667 (PMC7713972; doi:10.1002/ece3.6667)
Supplement: Supplementary file 1 — Supplementary Material [file ECE3-10-12727-s001.docx]

**Supplemental Background**

The genus *Rhagoletis* to which the cherry flies *R. indifferens* and *R. cingulata* belong is generally considered a model for ecological speciation-with-gene-flow (Bush, 1969, 1975; Berlocher & Feder, 2002; Drès & Mallet, 2002; Jiggins & Bridle, 2004). Certain species groups in the genus, such as the *R. pomonella* sibling species complex, have been proposed to radiate sympatrically by shifting and specializing on new host plants without geographic isolation (Bush, 1966, 1969; Berlocher, 2000; Xie *et al*., 2008). However, other species groups, like the *R. suavis* complex of walnut husk flies, all attack host plants in the genus *Juglans* (Bush, 1966). In contrast to *R. pomonella*, all the members of the *R. suavis* group are allopatric or partially parapatric due to secondary contact in their geographic distributions. In addition, all *R. suavis* group flies are morphologically distinct (Berlocher, 1976). Thus, it has been proposed that walnut husk flies diverged allopatrically through a combination of sexual selection and post-zygotic reproductive isolation (Bush, 1966; Berlocher, 1976; Glover *et al*., 2018).

The two focal species of the current study, *R. cingulata* and *R. indifferens*, are sister taxa in the *R. cingulata* species complex distributed across North America (Bush, 1966; Berlocher *et al*., 1993; Dowell & Penrose, 2012; Tadeo et al. 2015). The *R. cingulata* group has members that share some attributes with *R. pomonella* group flies, where sympatric ecological divergence is likely, and others with *R. suavis* group flies, where allopatric divergence has occurred (Bush & Smith, 1998). The species *R. cingulata* and *R. indifferens* seem more akin to *R. suavis* group flies. Although *R. cingulata* and *R. indifferens* do attack different species of cherries as their primary hosts (*R. cingulata* = black cherry, *Prunus serotina* Ehrh.; *R. indifferens* = bitter cherry, *P. emarginata* [Dougl. Ex Hook.] Eaton), both flies are known to use other cherry species in common as secondary hosts (Bush, 1966; Yee, 2008; Yee & Goughnour, 2005, 2008; Smith *et al*., 2014; Yee *et al*., 2015; Saint Jean *et al*., 2018). Thus, host affiliation is not likely to be a strong barrier to gene flow between *R. cingulata* and *R. indifferens*, if they were to co-occur in sympatry. Moreover, like *R. suavis* group flies, *R. cingulata* and *R. indifferens* vary morphologically (Bush, 1966). For example, in the eastern USA, most *R. cingulata* have an apical wing spot separated from the apical band by a hyaline area. However, in the Pacific Northwest, most *R. indifferens* lack the spot (Bush, 1966; Foote *et al.*, 1993). Studies of non-host related reproductive isolation between *R. cingulata* and *R. indifferens* (Hood *et al*., 2012) have indicated modest prezygotic isolation (isolation index = 0.27). It is possible that the apical wing spot is under sexual selection and contributes to the observed prezygotic isolation between *R. cingulata* in the East and *R. indifferens* in the Pacific Northwest but further mating experiments manipulating the spot are needed to confirm this hypothesis.

**Supplemental Methods**

*Microsatellites*

DNA was isolated and purified from adult head or whole-body fly tissue from a total of 364 flies from sites 1-15 for microsatellite analysis (Table 1) using PUREGENE extraction kits (Gentra Systems, Minneapolis, MN). Purified DNAs were transferred to 96-well plates for microsatellite PCR amplification and genotyping. Flies were genotyped for 21 dinucleotide repeat microsatellite loci. Twelve of the loci (WCFF07, WCFF024, WCFF031, WCFF057, WCFF061B, WCFF067, WCFF083, WCFF084A, WCFF086A, WCFF093, WCFF0105, WCFF0111) were developed for *R. indifferens* by Maxwell *et al*. (2009) and nine of the loci (P4, P27, P36, P37, P45, P50, P54, P71, P80) were developed originally for *R. pomonella* by Velez *et al*. (2006), but also cross amplify and are polymorphic in cherry flies. Forward and reverse primers and the conditions used to PCR amplify microsatellites are described in Maxwell *et al*. (2009, 2013) and Michel *et al*. (2010), respectively. The 21 microsatellites were chosen because they displayed no systematic evidence for heterozygote deficiency from Hardy-Weinberg equilibrium due to null alleles, as determined using MICRO-CHECKER (Van Oosterhout *et al*., 2004). Genotyping was performed on a Beckman-Coulter CEQ8000, as described in Saint Jean (2018). Microsatellite alleles were sized using the Fragment Analysis software provided by Beckman-Coulter (Brea, CA, USA). Size standards were included in each gel lane. In addition, for a subset of runs, three to four *R. cingulata* from sites in the eastern and southwest USA and Mexico, and *R. indifferens* from Woodland, WA (sites 2-3) were included to ensure that alleles were aligned and comparably scored.

*Genetic analysis of microsatellites*

An unrooted neighbor-joining genetic distance network for the 21 microsatellites based on Nei’s genetic distances (Nei, 1972) between populations was constructed using PowerMarker v3.25 (Liu & Mus, 2005). Bootstrap support for nodes were calculated based on 10,000 replicates across loci. Tests for isolation by distance (IBD) were done by regressing matrices of Nei’s genetic distances estimated for the microsatellites against log10 transformed values of the physical Euclidian distances between sites (in km) estimated using *geosphere* (R package version 1.3-11; Hijmans, 2014) by Mantel tests using *vegan* (R package version 2.2-1; Oksanen *et al*., 2015). Tests for evidence of genetic subdivision based on the microsatellites were performed using STRUCTURE v2.3.4 (Pritchard *et al*., 2000). Ten replicate runs were conducted, under the admixture model with correlated allele frequencies and no prior population information, for *K* values ranging from 1-15 with a burn-in of 500,000 iterations and 1,000,000 data collecting steps. The method of Evanno *et al*. (2005), based on the rate of change in the log probability of data between successive *K* values (delta *K*), was used to choose the number of clusters (subpopulations) that best fit the data (Kopelman *et al*., 2015).

Divergence times were estimated, based on the microsatellites, between cherry fly populations in the eastern USA (site 15; South Bend, IN) versus northern Mexico (site 11; Nuevo León), in the Pacific Northwest (site 4; Hood River, OR) versus southwest USA (site 7; Chiricahua Mountains, AZ), within the southwest USA between Arizona (site 7; Chiricahua Mountains) versus west Texas (site 8; Davis Mountains), and in southern Mexico (site 9; San Martín Texmelucan) versus northern Mexico (site 11; Nuevo León). Populations used for divergence estimates were selected from each region because they displayed the least amount of pairwise genetic divergence and/or were most geographically proximate, which made our estimates more conservative. Divergence times were derived using the Metropolis-coupled Markov Chain Monte Carlo (MCMC) sampling algorithm in the program IMa2p (Hey & Neilsen, 2004, Hey, 2010, Sethuraman & Hey, 2016). Posterior probability distributions for divergence times were generated using non-informative priors for ancestral population size (θ), migration rates between populations (*m*_1_ and *m*_2_), and time since divergence (*t*) in generations, which for *R. cingulata* and *R. indifferens* equals the number of years since separation, as these flies have one generation per year. To obtain an upper bound estimate for divergence time, *m*_1_ and *m*_2_ were both set to 0 under the assumption that there has been no gene flow following the separation of *R. cingulata* and *R. indifferens* into eastern, western, southwest, and Mexican subpopulations. We implemented the step-wise mutation model for microsatellite evolution and concentrated on mutation rates, congruent with other insects, of 1 × 10^-5^ and 6.3 × 10^-6^ per meiosis, the latter reflecting the estimate for *Drosophila* (Schug *et al*., 1997; Vazquez *et al*. 2000; Peccoud *et al*., 2009). We also assessed the consequences of higher mutation rates, ranging as high as 1×10^-3^ and including 1×10^-4^ (considered the mean for microsatellites; Estoup & Angers, 1998), on estimates of divergence times, focusing on two pairwise comparisons, South Bend, IN versus Nuevo León, MX and Hood River, OR versus Chiricahua Mountains, AZ. The 21 microsatellites genotyped conformed well to a dinucleotide repeat step-wise mutation model, with the exception that certain loci contained one or a few rare alleles differing from a strict 2 bp repeat pattern by a single nucleotide (see Table S4). In these cases, we adjusted the length of the allele, up or down, by one base pair. For each mutation rate and population pair analyzed, ten MCMC simulations were run for at least 1 × 10^7^ iterations, with a thinning period of 10,000, after an initial burn-in of 3 × 10^7^ iterations, using 200 chains and heating parameters ha = 0.99 and hb = 0.4. Results are reported for the ten pooled runs, which all converged to similar posterior distributions.

**Supplemental References**

Berlocher, S.H. 1976. The genetics of speciation in *Rhagoletis* (Diptera: Tephritidae). Ph.D. dissertation. Univ. of Texas at Austin, TX, USA.

Berlocher, S.H. 2000. Radiation and divergence in the *Rhagoletis pomonella* species group: inferences from allozymes. *Evolution* **54**: 543–557.

Berlocher, S.H. & Feder, J.L. 2002. Sympatric speciation in phytophagous insects: moving beyond controversy? *Annu. Rev. Entomol.* **47**: 773–815.

Berlocher, S.H., McPheron, B.A., Feder, J.L. & Bush, G.L. 1993. A revised phylogeny of the *Rhagoletis pomonella* (Diptera: Tephritidae) sibling species group. *Ann. Entomol. Soc. Am*. **86**: 716–727.

Bush, G.L. 1966. The taxonomy, cytology, and evolution of the genus *Rhagoletis* in North America (Diptera, Tephritidae). *Bull. Mus. Comp. Zool.* **134**: 431–562. Harvard University, Cambridge, MA, USA.

Bush, G.L. 1969. Sympatric Host Race Formation and Speciation in Frugivorous Flies of Genus *Rhagoletis* (Diptera, Tephritidae). *Evolution* **23**: 237-251.

Bush, G.L. 1975. Modes of animal speciation. *Annu. Rev. Ecol. Syst*. **6**: 339-364.

# Bush, G.L. & Smith, J.J. 1998. The genetics and ecology of sympatric speciation: A case study. *Res. Popul. Ecol.* 40: 175–187.

Dowell, R.V. & Penrose, R.L. 2012. Distribution and phenology of *Rhagoletis fausta* (Osten Sacken 1877) and *Rhagoletis indifferens* Curren 1932 (Diptera: Tephritidae) in California. *Pan-Pac. Entomol.* **88**:130-150.

Drès, M. & Mallet, J. 2002. Host races in plant-feeding insects and their importance in sympatric speciation. *Philos. Trans. R. Soc. London [Biol.]* **357**: 471–492.

Estoup A., & Angers, B. 1998. Microsatellites and minisatellites for molecular ecology: theoretical and empirical considerations. In: *Advances in Molecular Ecology* (G. Carvalho, ed.), pp. 55–86. NATO press, Amsterdam.

Evanno, G., Regnaut, S., & Goudet, J. 2005. Detecting the number of clusters of

individuals using the software STRUCTURE: a simulation study. *Mol.*

Foote, R.H., Blanc, F.L. & Norrbom, A.L. 1993. *Handbook of the Fruit Flies (Diptera: Tephritidae) of America North of Mexico.* Comstock Publishing Associates, Ithaca, New York.

Glover, M.M., Egan, S.P., Hood, G.R., Rull, J., Aluja, M. & Feder, J. L. 2018. Phylogeography of walnut-infesting *Rhagoletis suavis* (Diptera: Tephritidae) flies. *Insect Syst. Divers.*, **2**: 1.

Hey, J. 2010. Isolation with migration models for more than two populations. *Mol. Biol. Evol.* **27**: 905-920.

Hey, J. & Nielsen, R. 2004. Multilocus methods for estimating population sizes, migration rates and divergence time, with applications to the divergence of *Drosophila* *pseudoobscura* and *D. persimilis*. *Genetics* **167**: 747-760.

Hijmans, R.J. 2014. Geosphere: Spehrical Trigonometry. R. package version 1.3-11. http://CRAN.R-project.org/package=geosphere.

Hood, G.R., Egan, S.P. & Feder, J.L. 2012. Evidence for sexual isolation as a prezygotic barrier to gene flow between morphologically divergent species of *Rhagoletis* fruit flies. *Ecol. Entomol.* **37:** 521-528.

Jiggins, C.D. & Bridle, J.R. 2004. Speciation in the apple maggot fly: a blend of vintages? *Trends Ecol. Evol.* **19**: 111-114.

Kopelman, N.M., Mayzel, J., Jakobsson, M., Rosenberg, N.A. & Mayrose, I. 2015.

Clumpak: a program for identifying clustering modes and packaging population

structure inferences across *K. Mol. Ecol. Resour.* **15**: 1179-1191.

Liu, K.J. & Mus, S.V. 2005. PowerMarker: an integrated analysis environment for genetic

marker analysis*. Bioinformatics* **21**: 2128–2129.

Maxwell, S.A., Rasic, G. & Keyghobadi, N. 2009. Characterization of microsatellite loci for the western cherry fruit fly, *Rhagoletis indifferens* (Diptera: Tephritidae). *Mol. Ecol. Notes* **9**: 1025-1028.

**Maxwell, S.A., Thistlewood, H.M.A. & Keyghobadi, N. 2013. Population genetic structure of the western cherry fruit fly *Rhagoletis indifferens* (Diptera: Tephritidae) in British Columbia, Canada*. Agric. Forest Entomol.* 38: 823–835.**

Michel, A.P., Sim, S., Powell, T.H.Q., Taylor, M.S., Nosil, P. & Feder, J.L. 2010. Widespread genomic divergence during sympatric speciation. *PNAS* **107**: 9724–9729.

Nei, M. 1972. Genetic distance between populations. *Amer. Nat.* **106**: 283-292.

Oksanen, J., Guillaume Blanchet, F., Friendly, M., Kindt, R., Legendre, P., McGlinn, D. *et al*. 2015. Vegan: community ecology package. R package version 2.2-1. http://CRAN.R-project.org/package=vegan.

Peccoud, J., Simon, J.C., McLaughlin, H.J. & Moran, N. A. 2009. Post‐Pleistocene radiation of the pea aphid complex revealed by rapidly evolving endosymbionts. *PNAS* **106**: 16315–16320.

Pritchard, J.K., Stephens, M. & Donnelly, P. 2000. Inference of population structure using multilocus genotype data. *Genetics* **155**: 945-959.

Saint Jean, G., Hood, G.R., Egan, S.P., Powell, T.H.Q., Schuler, H., Doellman, M.M. *et al*. 2018. Limited Genetic Evidence for Host Plant-Related Differentiation in the Western Cherry Fruit Fly *Rhagoletis indifferens* (Diptera: Tephritidae). *Entomol. Exp. Appl.* doi: 10.1111/eea.12712.

Schug, M.D., Mackay, T.F. & Aquadro, C.F. 1997. Low mutation rates of microsatellite loci in *Drosophila melanogaster*. *Nat. Genet*. **15**: 99–102.

Sethuraman, A. & Hey, J. 2016. IMa2p – parallel MCMC and inference of ancient demography under the Isolation with migration (IM) model. *Mol. Ecol. Resour.* **16**: 206-215.

Smith, J.J., Powell, T.Q.H., Teixeira, L., Armstrong, W.O., McClowry, R. J., Isaacs, R. *et al.* 2014. Genetic structure of cherry fruit fly (*Rhagoletis Cingulata*) populations across managed, unmanaged, and natural habitats. *Entomologia exp. et. Applicata*, **150**: 157–165.

Tadeo, E., Feder, J.L., Egan, S.P., Schuler, H., Aluja, M. & Rull, J. 2015. Divergence and evolution of reproductive barriers among three allopatric populations of *Rhagoletis cingulata* across eastern North America and Mexico. *Entomol. Exp. Appl.* **156**: 301–311.

Van Oosterhout, C., Hutchinson, W.F., Wills, D.P.M. & Shirley, P. 2004. Micro-Checker: software for identifying and correcting genotyping errors in microsatellite data. *Mol. Ecol. Notes* **6**: 90-92.

Velez, S., Taylor, M.S., Noor, M.A.F., Lobo, N.F. & Feder, J.L. 2006. Isolation and characterization of microsatellite loci from the apple maggot fly, *Rhagoletis pomonella* (Diptera: Tephritidae). *Mol. Ecol. Notes* 6: 90-92.

Yee, W.L. & Goughnour, R. B. 2005. New hosts of western cherry fruit fly, *Rhagoletis indifferens* (Diptera: Tephritidae), and their relationship to life history characteristics of this fly. *Ann. Entomol. Soc. Amer*. **98**: 703-710.

Yee, W.L. 2008. Host plant use by apple maggot, western cherry fruit fly, and other *Rhagoletis* species (Diptera: Tephritidae) in central Washington state. *Pan-Pac. Entomol.* **84**: 163-178.

Yee, W.L. & Goughnour R. B. 2008. Host plant use and host records of apple maggot, western cherry fruit fly, and other *Rhagoletis* species (Diptera: Tephritidae) in western Washington state. *Pan-Pac. Entomol.* **84**: 179-193.

Yee, W.L., Lawrence, T.W., Hood, G.R. & Feder, J. L. 2015. New records of *Rhagoletis* Loew, 1862 (Diptera: Tephritidae) and their host plants in western Montana, U.S.A. *Pan-Pac. Entomol.* **91**: 39-57.

Xie, X., Michel, A.P., Schwarz, D., Rull, J., Velez, S., Forbes, A.A. *et al.* 2008. Radiation and divergence in the *Rhagoletis pomonella* species complex: inferences from DNA sequence data. *J. Evol. Biol.* **21**: 900-913.

**Figure S1**. Three basic modes of geographic distribution of populations during speciation. (a) Allopatric, with complete geographic isolation and no gene flow during population divergence; (b) Parapatric, with partial geographic isolation and some gene flow resulting in primary clines forming between diverging populations; and (c) Sympatric, with no geographic isolation and individuals of diverging populations within cruising range, resulting in the potential for gene flow. Populations initially diverge on the basis of adapting to alternate habitats or environments.

**Figure S2**. Three different mixed modes of divergence. (a) Allo-parapatric: ancestral population (orange) becomes geographically separated and isolated populations diverge in allopatry. The two isolates subsequently come into secondary contact and form secondary clines, with additional divergence evolving in parapatry; (b) Allo-para-allopatric: same as (a) except following secondary contact, populations again become geographically isolated, with additional divergence evolving in allopatry; (c) Para-allopatric: ancestral population initially diverges in parapatry forming primary clines. Subsequently, populations become geographically isolated, with additional divergence evolving in allopatry.

**Figure S3**. Relationship between microsatellite mutation rate assumed (range 6.3 x 10^-5^ to 1 x 10^-3^; log scale) and the IMa2p estimated divergence times in years (log scale) between pairs of cherry fly populations in South Bend, IN and Nuevo León, Mexico (solid line) and Hood River, OR and the Chiricahua Mountains, AZ (dashed line).


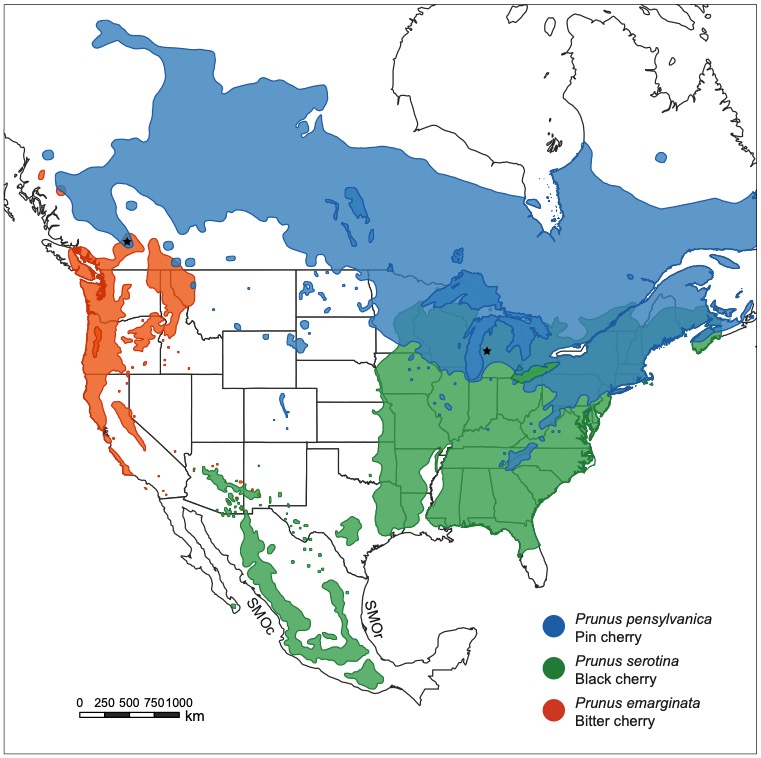


**Figure S4**. Distribution of *Prunus pensylvanica*, pin cherry, in blue superimposed on that for *P. serotina*, black cherry, in green and *P. emarginata*, bitter cherry, in orange. SMOr = Sierra Madre Oriental; SMOc = Sierra Madre Occidental Mountains. Stars denote collections of *R. indifferens* and *R. cingulata* from pin cherry in British Columbia, Canada (Maxwell et al., 2013) and Michigan (Bush, 1966), respectively. Note the potential for a past distributional ring for cherry-infesting flies formed by black, pin, and bitter cherry host plant species around the northern and central plains and Great Basin regions in North America.

**Table S1.** Site designations (#), locations, latitude and longitude, number of flies genotyped for microsatellites (n-msat), and number of flies sequenced for mtDNA (n-mtDNA). Host for sites 1-6 = *Prunus emarginata* (bitter cherry), sites 7-17 = *P. serotina* (black cherry). Sites 16 and 17 were sequenced for mtDNA only.

| # | Location | Latitude, Longitude | | n-msat | n-mtDNA | |
| --- | --- | --- | --- | --- | --- | --- |
|  |  |  | |  |  | |
| 1 | Salmon Arm, British Columbia, Canada | 50°54'54", -119°21'26" | | 19 | -- | |
|  |  |  | |  |  | |
| 2 | L. Kalama Road, Woodland, Washington | 45°56'24", -122°40'41" | | 43 | -- | |
|  |  |  | |  |  | |
| 3 | Lewis River, Woodland, Washington | 45°56'20", -122°38'5" | | 34 | 5 | |
|  |  |  | |  |  | |
| 4 | Trout Creek Rd, Hood River, Oregon | 45°32'10", -121°37'14" | | 16 | -- | |
|  |  |  | |  |  | |
| 5 | Ronald, Kittitas County, Washington | 47°23'50", -121°02'67'' | | 48 | -- | |
|  |  |  | |  |  | |
| 6 | Somers, Montana | 48°05'24", -114°13'48" | | 11 | 5 | |
|  |  |  | |  |  | |
| 7 | Chiricahua Mountains, Arizona | 31°55'44", -109°5'15'" | | 24 | 4 | |
|  |  |  | |  |  | |
| 8 | Davis Mountains, Texas | 30°38'47", -104°01'08" | | 23 | 4 | |
|  |  |  | |  |  | |
| 9 | San Martín Texmelucan, Mexico | 19°16' 20", -98°25'03" | | 24 | 1 | |
|  |  |  | |  |  | |
| 10 | Huamantla, Tlaxcala, Mexico | 19°18'54", -97°52'28" | | 24 | 13 | |
|  |  |  | |  |  | |
| 11 | Nuevo León, Mexico | 25°48'48", -100°22'02" | | 24 | 8 | |
|  |  |  | |  |  | |
| 12 | Los Lirios, Coahuila, Mexico | 25°20'58", -100°17'46" | | 24 | -- | |
|  |  |  | |  |  | |
| 13 | Live Oak, Suwannee County, Florida | 30°22'06", -83°14'38" | | 14 | 6 | |
|  |  |  | |  |  | |
| 14 | Green Creek, Cape May, New Jersey | 39°02'11", -74°54'05" | | 16 | 4 | |
|  |  |  | |  |  | |
| 15 | South Bend, Indiana | 41°45'28", -86°12'4" | | 20 | 13 | |
|  |  |  | |  |  | |
| 16 | Urbana, Illinois | 40° 6'17", -88°11'58" | | -- | 11 | |
|  |  |  |  | |  |  |
| 17 | Gila Cliff Dwelling Mon., New Mexico | 33°10'44", -108°12'14" | -- | | 15 |  |

**Table S2.** Pairwise estimates of *D_Jost_* between all 15 populations, based on 21 microsatellite loci. See Table S1 for full list of designations and descriptions of study sites and Figure 1a for a map of collecting sites.

|  | 1 | 2 | 3 | 4 | 5 | 6 | 7 | 8 | 9 | 10 | 11 | 12 | 13 | 14 |
| --- | --- | --- | --- | --- | --- | --- | --- | --- | --- | --- | --- | --- | --- | --- |
| 2 | 0.0239 |  |  |  |  |  |  |  |  |  |  |  |  |  |
| 3 | 0.0054 | 0.0034 |  |  |  |  |  |  |  |  |  |  |  |  |
| 4 | 0.0043 | 0.0021 | 0.0006 |  |  |  |  |  |  |  |  |  |  |  |
| 5 | 0.0129 | 0.0165 | 0.0086 | 0.0086 |  |  |  |  |  |  |  |  |  |  |
| 6 | 0.0118 | 0.0064 | 0.0066 | 0.0094 | 0.0067 |  |  |  |  |  |  |  |  |  |
| 7 | 0.0627 | 0.0492 | 0.0521 | 0.0491 | 0.0503 | 0.0302 |  |  |  |  |  |  |  |  |
| 8 | 0.1693 | 0.1717 | 0.1564 | 0.1547 | 0.1524 | 0.1599 | 0.0833 |  |  |  |  |  |  |  |
| 9 | 0.2851 | 0.2873 | 0.2697 | 0.2802 | 0.3037 | 0.2782 | 0.2619 | 0.1858 |  |  |  |  |  |  |
| 10 | 0.2656 | 0.2868 | 0.2625 | 0.2632 | 0.2885 | 0.2709 | 0.2587 | 0.1721 | 0.0354 |  |  |  |  |  |
| 11 | 0.1682 | 0.1536 | 0.1569 | 0.1567 | 0.1430 | 0.1701 | 0.1369 | 0.0337 | 0.1658 | 0.1258 |  |  |  |  |
| 12 | 0.2276 | 0.2093 | 0.2027 | 0.2059 | 0.1814 | 0.2302 | 0.1970 | 0.0703 | 0.1313 | 0.1677 | 0.0349 |  |  |  |
| 13 | 0.3507 | 0.3254 | 0.3176 | 0.3049 | 0.3055 | 0.3454 | 0.2719 | 0.1100 | 0.2036 | 0.1620 | 0.0926 | 0.1417 |  |  |
| 14 | 0.3813 | 0.3537 | 0.3475 | 0.3319 | 0.3367 | 0.3773 | 0.2667 | 0.1164 | 0.2433 | 0.2165 | 0.1111 | 0.1505 | 0.0000 |  |
| 15 | 0.3051 | 0.2798 | 0.2857 | 0.2738 | 0.2766 | 0.3183 | 0.2503 | 0.1077 | 0.3254 | 0.2452 | 0.0851 | 0.1458 | 0.0103 | 0.0154 |

**Table S3.** Microsatellite allele frequencies for 15 cherry-infesting populations of *Rhagoletis* surveyed across North America. Populations 1-6 = bitter cherry-infesting populations of *R. indifferens* in the Pacific Northwest (1 = Salmon Arm, British Columbia, Canada; 2 = Little Kalama Road, Woodland, Washington, USA; 3 = Lewis River, Woodland, Washington, U.S.; 4 = Trout Creek Rd, Mt. Hood, Oregon, USA; 5 = Ronald, Kittitas County, Washington, U.S.; 6 = Somers, Montana, USA); Populations 7, 8 = black cherry-infesting populations of *R. cingulata* in the southwest USA (7 = Chiricahua Mountains, Portal, Arizona, USA; 8 = Davis Mountains, Texas, USA); Populations 9-12 = black cherry-infesting populations of *R. cingulata* in Mexico: (9 = San Martín Texmelucan, MX; 10 = Huamantla, Tlaxcala, MX; 11 = Nuevo León, MX; 12 = Los Lirios, Coahuila, MX); Populations 13-15 = black cherry-infesting populations *of R. cingulata* in the eastern USA (13 = Live Oak, Suwannee County, Florida, USA; 14 = Green Creek, Cape May, New Jersey, USA.; 15 = South Bend, Indiana, USA). Orange boxes indicate alleles shared between Pacific Northwest sites with southwestern USA and Mexican sites; Green boxes indicate alleles shared between eastern USA sites with southwestern USA and Mexican sites; Blue boxes indicate alleles unique to southwestern USA and Mexican sites; Yellow boxes indicate alleles unique to Pacific Northwest sites; Red boxes indicate alleles unique to the eastern USA sites. Sample sizes (number of flies scored) are given in the bottom row for each microsatellite.

See separate .xlsx file (RhagoletisBiogeoTableS3).
